# Intrinsic Chiroptical Evolution in Perovskite Nanocrystals

**DOI:** 10.1021/acs.jpclett.5c03687

**Published:** 2026-01-30

**Authors:** Pengbo Ding, Dezhang Chen, Mohsen Tamtaji, GuanHua Chen, Liang Guo, Jonathan E. Halpert

**Affiliations:** † Department of Chemistry, 58207 The Hong Kong University of Science and Technology, Hong Kong SAR 999077, China; ‡ Department of Mechanical and Energy Engineering, Southern University of Science and Technology, Shenzhen 518055, China; § Hong Kong Quantum AI Lab Limited, Pak Shek Kok, Hong Kong SAR 999077, China; ∥ Department of Chemistry, The University of Hong Kong, Hong Kong SAR 999077, China; ⊥ SUSTech Energy Institute for Carbon Neutrality, Southern University of Science and Technology, 518055 Shenzhen, China

## Abstract

Here, we observed chiroptical evolution during the synthesis of perovskite nanocrystals (NCs). Using a steric-delayed nucleation strategy, we slowed the ripening of NCs and observed changes in the circular dichroism (CD) signal of perovskite NCs. Two chiroptical evolution events occurred during NC formation under different conditions, attributed to ligand displacement and NC self-assembly, respectively. Surface characterization and computational studies demonstrate that the ligands initially bind to the NC surface and dissociate during the ripening stage, offering the time window to observe and enhance the intrinsic chirality. Our findings provide a new strategy for studying and designing intrinsically chiral perovskite nanostructures.

## Graphical abstract

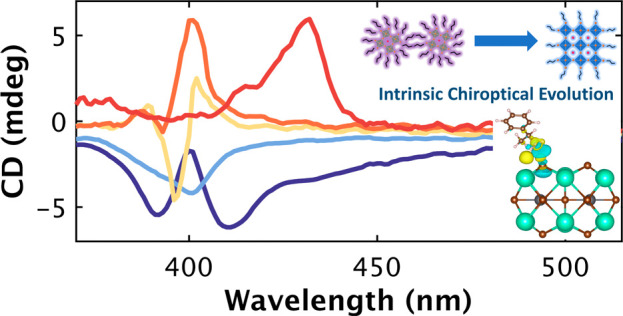

The study of chiral semiconducting nanomaterials has generated much interest due to their ability to interact with circularly polarized light and exhibit enantioselective properties.^1^ This makes them highly promising for applications in biosensing,^2^ asymmetric catalysis,^3^ optical devices,^4^ and spintronics.^5^ The chirality in nanomaterials arises from several mechanisms, including a chiral lattice or defects within the core (intrinsic chirality),^6,7^ a chiral surface structure of the core,^8^ chiral assembly,^9^ and polarization effects within the crystal lattice.^10^ Of these, intrinsic chirality is particularly compelling because it emerges solely from the atomic arrangement in the lattice, eliminating the need for external chiral molecules. Inorganic halide perovskite (CsPbX_3_, where X = Cl, Br, I) nanocrystals (NCs) have garnered significant attention in optoelectronic applications due to their defect tolerance properties.^11,12^ Despite significant progress in inducing chirality with chiral ligands,^4,13,14^ investigations into the origin of their intrinsic chiroptical properties remain limited.^15^ The theoretical and experimental studies of perovskite nanostructures always examined static, fixed structures,^16–18^ offering limited insights into the dynamic processes and mechanisms underlying the chiral structure formation. A critical challenge persists in understanding and developing methodologies to control and enhance the intrinsic chirality in these perovskite NCs.

Here, we observed in situ the evolution of the circular dichroism (CD) signal in perovskite NCs. This is achieved by adding the steric ligand during the synthesis, which delays the NC growth time to 30 min and increases the intrinsic chirality of NCs. We also studied two chiroptical inversion events, in which the CD signal flips its sign during synthesis. With the help of time-dependent density functional theory (TD-DFT) simulation, we attribute these two inversions to the ligand displacement during the NC ripening and from a screw dislocation during the NC self-assembly, respectively. Combining surface characterizations with DFT calculations, we reveal that the steric ligand serves as a transient surface modulator rather than the binding ligand, which repulses adjacent seeds during nucleation, delays the growth dynamics and enhances the formation of an intrinsic chiral lattice.

We synthesized CsPbBr_3_ NCs using a modified ligand-assisted reprecipitation (LARP) method,^19^ incorporating racemic methylbenzylammonium bromide (MBABr) as a sterically hindered ligand. While the pristine LARP synthesis formed quantum dots (QDs) right after the precursor injection, the addition of MBA delayed the growth dynamics and yielded smaller nanoclusters (NCLs) at early stages (Figure 1a). These NCLs, characterized by purple emission with an absorption peak at 400 nm, are consistent with CsPbBr_3_ NCLs in the previous study^20^ and contrasted with the blue-emitting QDs (Figure 1b). These NCLs are unstable and undergo rapid ripening within 30 min, forming quantum dots (QDs) with absorption and photoluminescence (PL) features comparable to those of the pristine QDs. Given that the NCLs exhibit optical characteristics similar to those of 2D perovskite monolayers,^20,21^ we further ruled out the presence of 2D monolayer fragments based on the absence of any extended 2D morphologies in the TEM images (Figure S2).

**Figure 1:**
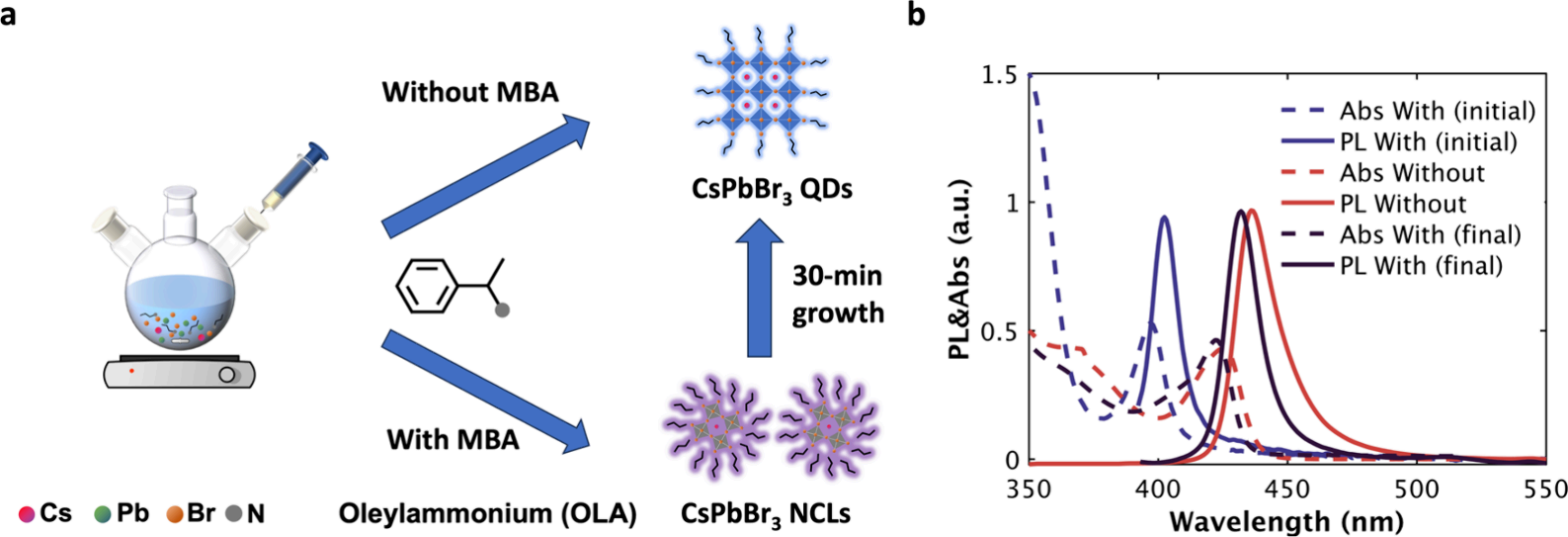
(a) Schematic representation of the modified sterically controlled LARP method to synthesize CsPbBr_3_ QDs and NCLs, in which NCLs grow to QDs over a period of 30 min. (b) UV–vis absorption and PL spectra of NCs with and without MBA ligands.

The delayed growth process enables us to gain mechanistic insight into the evolution and enhancement of chiroptical properties. Therefore, we conducted in situ absorption and CD measurements at temperatures from 30 to 90 °C (Figure 2a,b). The resulting NCs exhibit PL from purple to blue to green (Figure 2c), corresponding to larger NC sizes and reduced quantum confinement at higher temperatures. The larger size NC also exhibits lower *g*_CD_ (Figures S2 and S3), because the intrinsic chirality is less significant in the weakly confined regime.^22^ Control syntheses performed without MBABr also yield CsPbBr_3_ NCs with measurable CD signals, but with lower intensity than that in the MBA-assisted case (Figure S3), indicating that the perovskite lattice can exhibit intrinsic chiroptical activity while MBA amplifies and reshapes this underlying response via kinetic regulation. For the 50 and 90 °C samples, the absorption spectra and the CD signals remain unchanged during the entire 30 min reaction, indicating that the final structure is formed at the beginning of the reaction. However, the 30 and 70 °C samples exhibit distinct chiroptical inversion events during evolution.

**Figure 2:**
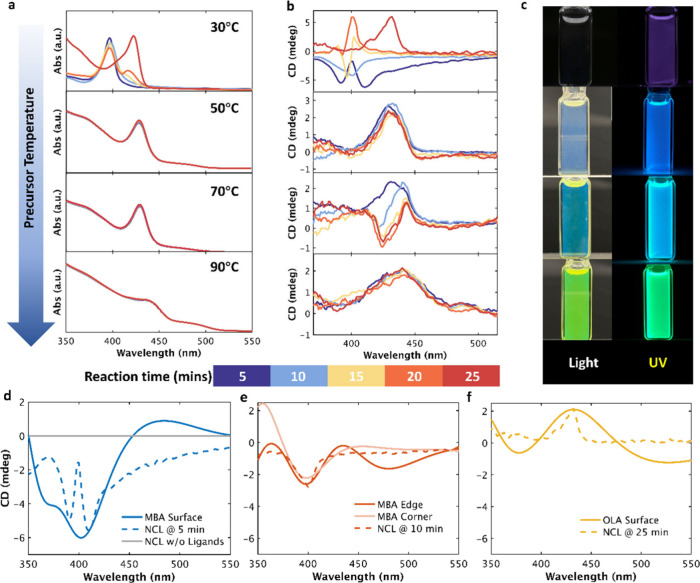
In-situ optical characterization of CsPbBr_3_ NC evolution synthesized under different temperatures. (a) Absorption spectra, (b) CD spectra, (c) PL photos of 30 °C, 50 °C, 70 °C, and 90 °C samples. Experimental (dashed) and TD-DFT-calculated (solid) CD spectra for CsPbBr_3_ NCLs with different ligand binding modes. (d) MBA bound to surface; (e) MBA bound to edge and corner sites; (f) OLA bound to surface. For visual clarity, a fixed *y*-axis scale was used to highlight the temporal evolution of the CD spectral shape from the same reaction batch.

For the 30 °C samples, the absorption spectra show a redshift in the excitonic peak from 395 to 425 nm over 25 min, reflecting the transition from small NCLs to QDs. Simultaneously, the CD spectra exhibit a pronounced inversion from negative to positive: an initially strong negative signal with split peaks (5 min) gradually diminishes and then reverses to a positive CD signal near 430 nm (25 min). In addition, faster scan CD measurements by narrowing the spectral range reveal no additional transient features (Figure S4). We conducted TD-DFT calculations (Figure 2d–f) to theoretically investigate ligand-induced chirality. Without surface binding ligands, there is no chiroptical response observed in NCLs, confirming that the chirality originates from the lattice-ligand interaction. When a single MBA was bound to the NCs’ surface, the simulated NCs exhibited a strong negative signal at around 408 nm with a negative rotational strength, consistent with the experimental result at 5 min (Figure 2d). When the MBA was bound to NC edge or corner sites, the signal at 408 nm (Figure 2e) was less intense, and corresponded to the evolution process at 10 min. When the OLA was bound to the NC surface, a positive signal at 435 nm was observed, consistent with the experimental results at the end of the NC evolution (Figure 2f). Therefore, we attribute this inversion to a ligand exchange process, in which OLA ligands subsequently replace the initial binding of MBA ligands as the particles grow and ripen. Experimentally, the synthesized QDs exhibit a distorted lattice, as shown in the TEM images (Figure S5). The initial CD spectrum shows two closely spaced negative bands, which we attribute to ligand-induced lattice distortion rather than to sample heterogeneity, since both absorption and PL spectra exhibit a single excitonic peak.

For the 70 °C sample, the absorption spectra (Figure 2a) show negligible spectral shift over time, but the CD spectra evolve into a distinct bisignate feature near the excitonic peak (430 nm) after 10 min. We attribut this chiroptical behavior to the self-assembly of QDs into twisted NWs, as confirmed by TEM images and diameter comparisons.^23,24^ These NWs exhibit asymmetric lattice structures (Figure S6), consistent with previously reported mechanisms for intrinsic chirality in 1D perovskite architectures.^24^ The resulting chiral coupling among adjacent QDs gives rise to excitonic interactions that manifest as the Cotton effect. The CD signal stopped evolving after 15 min, and the bisignate feature persisted for over 8 h (Figure S7), supporting the conclusion that the signal arises from the stable NW assembly and is consistent with previous reports.^24^ To eliminate the chirality transfer from chiral MBA molecule, we synthesized QDs using either racemic or enantiopure MBABr with nearly identical CD signals (Figure S8), indicating that MBABr’s effect is primarily steric rather than arising from enantioselective binding.^25^ Also, the CD features were reproducibly observed across three independent batches (Figure S9), confirming their robustness under identical synthesis conditions.

We conducted a combination of spectroscopic characterizations and theoretical modeling to elucidate the role of MBA ligands during NC formation. FTIR spectra (Figure 3a) reveal the characteristic aromatic C–H stretches of pure MBABr, which are absent in both NC samples, suggesting that MBA does not remain bound to the surface after synthesis. This conclusion is further supported by proton NMR analysis (Figure 3b,c). The sharp MBABr peaks observed in the precursor solution, particularly at ∼7.35 and 7.50 ppm in the aromatic region, disappear in the final products. These FTIR and NMR spectra were recorded on purified NCs after washing cycles, so free MBA ligands were removed with the supernatant. The absence of MBA signatures therefore indicates that MBA does not remain as a major surface component in the final product.

**Figure 3:**
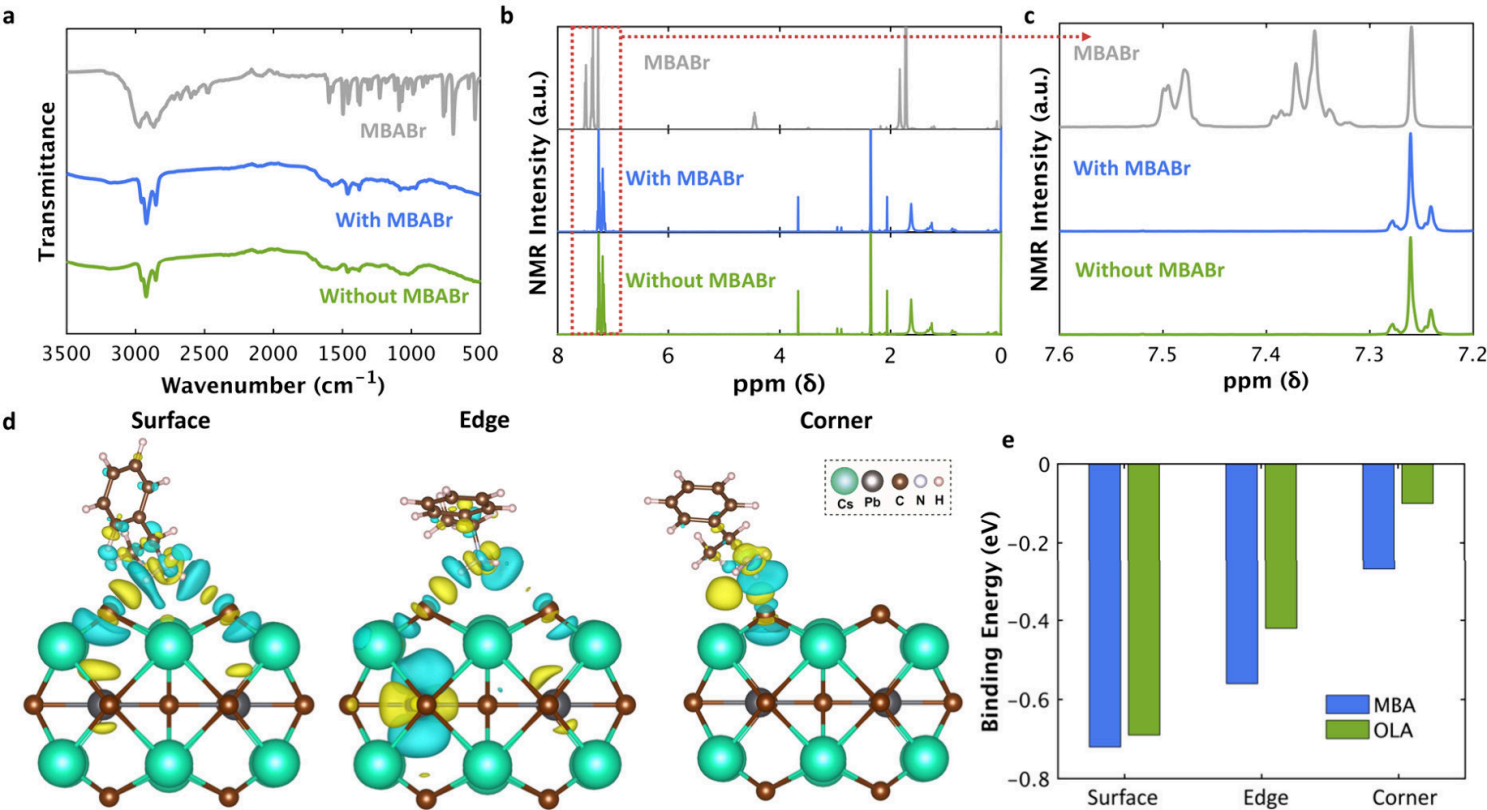
Experimental and computational surface binding analysis. (a) FTIR spectra and (b) NMR spectra (c) zoomed-in NMR region of MBABr salt, the QDs synthesized with or without MBABr. (d) DFT simulations showing charge transfer between MBA and CsPbBr_3_ binding at the surface, edge, and corner sites. Yellow and blue colors represent electron availability and deficiency with an isosurface value of 0.0012 e Å^–3^. (e) Binding energy plot comparing MBA with OLA.

To gain deeper insight into the binding behavior, we performed DFT calculations to examine the interactions between the MBA and OLA and representative CsPbBr_3_ surface states. Given the surface-dominated nature of early stage NCLs, we modeled a 2 × 2 × 2 [PbBr_6_]^4–^ octahedral lattice with surface, edge, and corner binding states (Figure 3d) to simulate the binding environment. In larger NCs (*n* > 5, where n is the number of octahedral units per edge), the number of surface sites dominates, while the number of corner sites becomes significant in ultrasmall NCs (*n* = 2). Binding energy calculations (Figure 3e) show that MBA exhibits much stronger adsorption than OLA at corner sites (−0.27 eV vs −0.10 eV) but comparable adsorption at surface sites (−0.72 eV vs −0.69 eV). This results in more MBA ligands being attached to the [PbBr_6_]^4–^ monomer, impeding their coalescence, and slowing NCL-to-QD transitions. Despite MBA’s stronger binding to corner sites compared to that of OLA, its binding energy is weaker than that on QD surfaces (−0.69 eV). Due to steric hindrance from bulky phenyl rings, MBA adsorption on NC surfaces is energetically unfavorable,^26^ whereas OLA forms dense surface layers.^27^ As a result, MBA eventually dissociates and is replaced by OLA, allowing the growth of NCLs into QDs.^28,29^ This conclusion is also consistent with the TD-DFT results that the transition of ligands drives the chiroptical inversion process. The specific geometry of MBA, with an α-methyl substituent and a phenyl ring at a moderate distance from the ammonium group, allows it to form MBA–PbBr_2_ complexes that slow nucleation without driving the system into 2D layered phases^26^ or binding strongly enough to generate persistent ligand-bound chirality (Figure S10). We further conducted MBA concentration-dependent synthesis to study the growth mechanism (Figures S11 and S12). The results show that excessive MBA hinders the formation of larger NCs even at high temperatures, while insufficient MBA fails to stabilize NCLs at early stages. These findings confirm that MBA preferentially binds to small NCLs, effectively slowing down the ripening process into QDs. We therefore attribute the spatially delayed nucleation primarily to the formation of metastable intermediate clusters that buffer precursor supersaturation and slow CsPbBr_3_ nucleation.

To conclude, we successfully observed the intrinsic chiroptical evolution in perovskite NCs. By delaying the growth dynamics and varying the reaction temperature, we observed and explained two chiroptical inversion events, in which the CD signal flips sign during the reaction. Experiments and DFT simulations reveal that MBA acts as a transient ligand, binding weakly to [PbBr_6_]^4–^ octahedra at early stages to slow down the growth and induce lattice distortions. When the MBA dissociates and is replaced by OLA ligands, the CD signal inverts, and stronger intrinsic chirality is observed. Our work reveals the image of chiroptical evolution in perovskite NCs, offering a blueprint for future studies of transient phenomena in dynamic nanostructure evolution.

## Supplementary Material



## Notes

Experimental section, computational details, supplementary figures (structures, TEM, UV–vis spectra, CD spectra of the CsPbBr_3_ NCs with and without MBA ligands, CD spectra for NCs with racemic and alkyl ligands, batch difference and stability test, PL spectra), concentration-dependent analysis (PDF)
