# Supplementary material for: Intrinsic Chiroptical Evolution in Perovskite Nanocrystals
Source: J Phys Chem Lett. 2026 Jan 30;17(6):1793–7. doi: 10.1021/acs.jpclett.5c03687 (PMC12908148; doi:10.1021/acs.jpclett.5c03687)
Supplement: Supplementary file 1 [file jz5c03687_si_001.pdf]

Supporting Information for Publication

Intrinsic Chiroptical Evolution in Perovskite Nanocrystals

Pengbo Ding,[1,2#] Dezhang Chen,[1#] Mohsen Tamtaji,[3] GuanHua Chen,[3,4*] Liang Guo,[2,5*] Jonathan E. Halpert[1*]

[1]Department of Chemistry, The Hong Kong University of Science and Technology, Hong Kong SAR 999077, China,

[2]Department of Mechanical and Energy Engineering, Southern University of Science and Technology, Shenzhen 518055, China

[3]Hong Kong Quantum AI Lab Limited, Pak Shek Kok, Hong Kong SAR 999077, China

[4]Department of Chemistry, The University of Hong Kong, Hong Kong SAR 999077, China

[5]SUSTech Energy Institute for Carbon Neutrality, Southern University of Science and Technology, 518055 Shenzhen, China

[*]Corresponding Author:

GuanHua Chen (ghc@everest.hku.hk); Liang Guo (guol3@sustech.edu.cn); Jonathan E. Halpert (jhalpert@ust.hk)

[#]These authors contributed equally: Pengbo Ding, Dezhang Chen.

Keywords: chirality, perovskite, nanocrystals, ligands

Table of Contents

Experimental Section -- 3

Computational Details -- 5

Supplementary Figures -- 7

MBABr Concentration-Dependent Analysis -- 13

Reference -- 16

Experimental Section

Materials: Cesium bromide (CsBr, 99.9%, Sigma-Aldrich), lead bromide ($PbBr_2$, 99.9%, Sigma–Aldrich), oleic acid (OA, 90%, Sigma–Aldrich), oleylamine (OLA, 95%, Sigma–Aldrich), N, N-dimethylformamide (DMF, anhydrous, 99.8%, Sigma–Aldrich), toluene (anhydrous, 99.8%, Sigma–Aldrich), 4-methylbenzylammonium bromide (MBABr, 99%, Xian Yuri Solar Co.) All chemicals were used as received without further purification.

Synthesis of $CsPbBr_3$ NCs: In typical synthesis of $CsPbBr_3$ NCs using a modified ligand-assisted reprecipitation (LARP) method, 0.5 mmol cesium bromide (CsBr), 2 mmol lead bromide ($PbBr_2$), and varying amounts of racemic methylbenzylammonium bromide (rac-MBABr) (0 mg, 30 mg, 60 mg, and 90 mg) were added to a 25 mL three-neck flask containing 1.8 mL oleic acid (OA), 0.6 mL oleylamine (OLA), and 10 mL N,N-dimethylformamide (DMF). An excess amount of $PbBr_2$ was employed to slow the growth process, controlling the formation of $CsPbBr_3$. Rac-MBABr was introduced as sterically hindered ligands to induce octahedral distortion, promoting chiral lattice structures or shapes.[1] To investigate nucleation and growth dynamics, we varied the precursor temperature (30–90 °C) at a fixed concentration, allowing us to explore the growth processes of different $CsPbBr_3$ nanostructures under similar reaction conditions. After stirring for 30 seconds, the mixture was heated to target temperature (30℃, 50℃, 70℃, and 90℃), stirred at the target temperature for additional 30 seconds, and then 1 mL of the precursor solution was injected into 50 mL of toluene under vigorous stirring. The resulting solution was stirred for 30 second and the in-situ measurements were conducted. For ex-situ measurements (TEM, FTIR, NMR), nanostructures were isolated by adding antisolvent (methyl acetate, volume ratio 3:1) and

centrifuge at 15000 rpm for 5 minutes. The precipitate was obtained and suspended in toluene. For NMR measurements, the precipitate was suspended in $CDCl_3$.

Characterization: Ultraviolet-visible (UV–vis) absorption spectra were recorded using a Shimadzu UV3600 spectrometer. TEM/HRTEM images were obtained on a JEOL JEM-2100 with an acceleration voltage of 200 kV. Samples were prepared by drop-casting dilute NC dispersions onto carbon-coated copper grids. NMR measurements were conducted on a BRUKER AVII 400 NMR instrument. The steady-state PL spectra were recorded with a 600 μm reflection probe (R600-7-UV-125F) coupled with a QE Pro spectrometer (Ocean Optics) using a 365 nm LED as the exciting source.

CD measurements: CD measurements were carried out on a Chirascan V100 (Applied Photophysics) circular dichroism spectrometer with the cuvette placed in the beam path. All spectra obtained were averages of three scans except for the time-resolved spectra. The time-resolved CD spectra had been smoothed by 5-point averaging for a clearer display of the evolution. CD spectra were recorded from 230 to 600 nm with a 1 nm resolution, 1 sec integration time per dot, and the data were presented as the raw CD signal.

Computational Details

DFT calculation details: Spin-polarized density functional theory (DFT) calculations were performed using the Vienna ab initio Simulation Package (VASP, version 6.1.0) code[2] with Perdew-Burke-Ernzerhof (PBE)[3] functional to calculate the binding energy and charge transfer between $CsPbBr_3$ and MBA or $CsPbBr_3$ and OLA (Figure S1). A plane-wave cutoff energy of 450 eV is used. As shown in Figure S1, a small cube of $CsPbBr_3$ was constructed inside a box with the size of 22.31Å×22.31Å×22.31Å with either an MBA or OLA molecule positioned on its surface (Figure S1). To consider the vdW interactions (London dispersion forces), the D3 correction was utilized with the Becke-Johnson damping function.[4] VASPsol was used to account for implicit solvation. The Brillouin zone for structural relaxation and self-consistent calculations was sampled using a 1×1×1 k-point grid based on the Monkhorst-Pack scheme. [5] During the structural relaxation, the atoms for $CsPbBr_3$ were frozen and MBA or OLA molecules were allowed to relax on $CsPbBr_3$. The convergence criteria for the electronic structure energy and force on each atom were set to be 10^{-5} eV and 0.02 eV/Å, respectively. For self-consistent calculations, the convergence criterion for the electronic structure energy was set to be 10^{-6} eV.

TD-DFT calculation details: Time-dependent density functional theory (TD-DFT) calculations used the Coulomb-attenuating method combined with the Becke 3-parameter, Lee, Yang, and Parr hybrid functional (CAM-B3LYP/LANL2DZ)[6] for frequency and excitation calculations of the relaxed MBA and OLA structures obtained from the aforementioned DFT calculations. We used the solvation model density (SMD) as a universal continuum solvation model to include solvent effects.[7] In addition, we calculated the energy levels of the singlet and triplet excitons, considering

a total of 30 states, to predict the CD, based on the optimized ground state geometries.[8] We also applied the DFT-D3 method to include van der Waals attraction (London dispersion) interactions.[9,10] All the TD-DFT calculations were performed using Gaussian 16 software, and visualizations were performed using GaussView 6.0.16.[11] In the TD-DFT cluster calculations (Figure 2d–f), we use a single MBA enantiomer (R-MBA) as a representative ligand to introduce local asymmetry; the resulting CD sign is governed primarily by ligand-induced distortion of the inorganic framework rather than by the absolute configuration of MBA. Consistent with this, racemic, R-, and S-MBA yield indistinguishable experimental CD spectra under the same conditions (Figure S8). The sign of the CD peak is determined by the rotational strength, defined as the imaginary part of the scalar product between the electric dipole transition moment and the magnetic dipole transition moment for excited states. Positive values of rotational strength correspond to positive CD signals, while negative values correspond to negative CD signals.

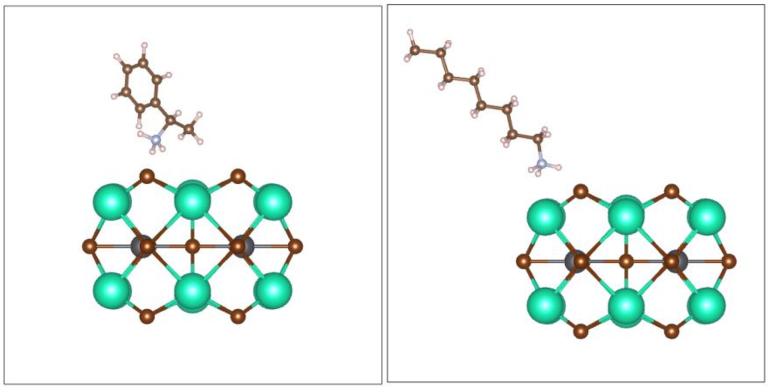

Figure S1. The structure of a small cube of $CsPbBr_3$ inside a box with the size of 22.31Å×22.31Å×22.31Å with either an MBA (left panel) or OLA (right panel) molecule positioned on its surface.

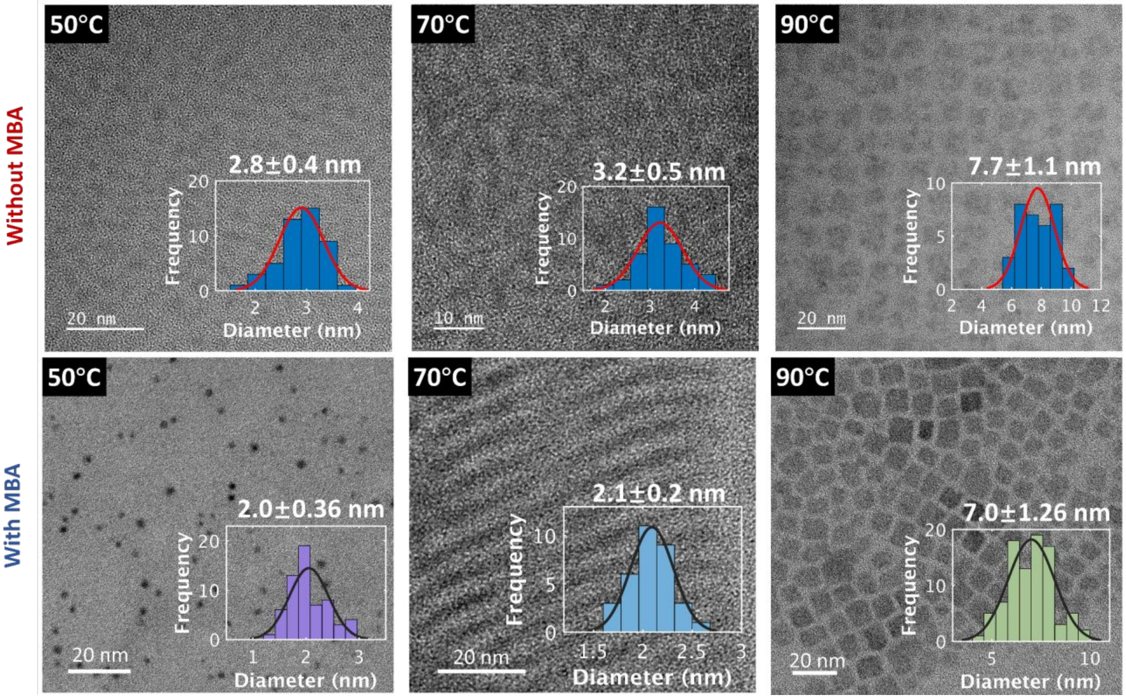

Figure S2. HRTEM of the $CsPbBr_3$ NCs synthesized under different temperatures. The rapid evolution of 30°C sample makes the TEM image and accurate size counting impossible.

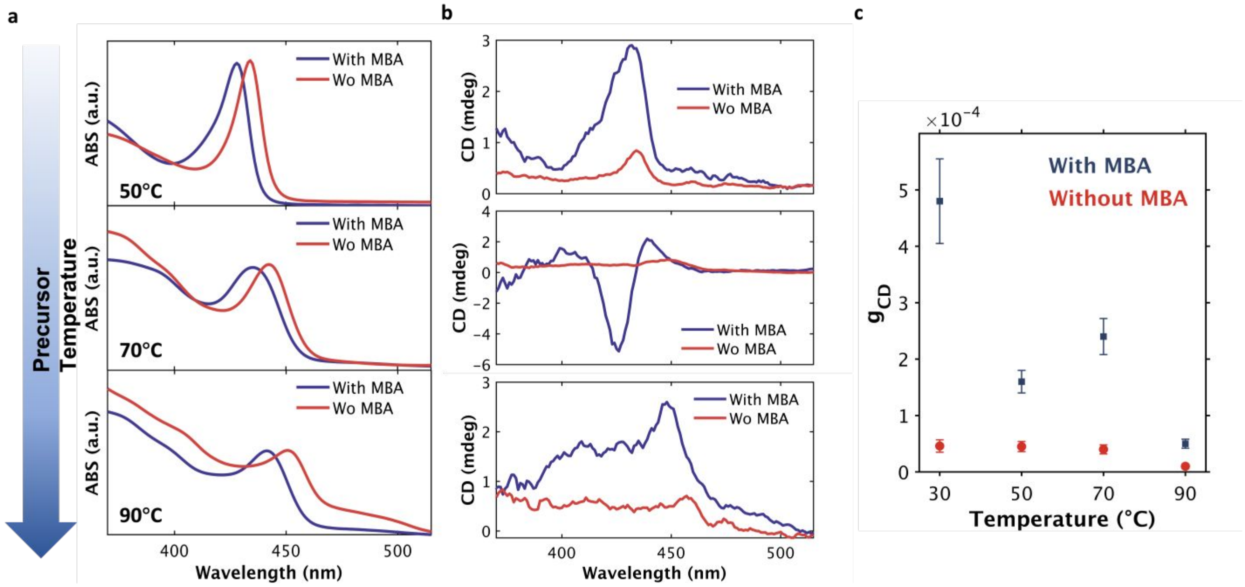

Figure S3. Comparison of (a) UV-vis, (b) CD spectra, and (c) g_{CD} of NCs with or without the ligand additive. Error bars represent the standard deviation calculated by three different batches.

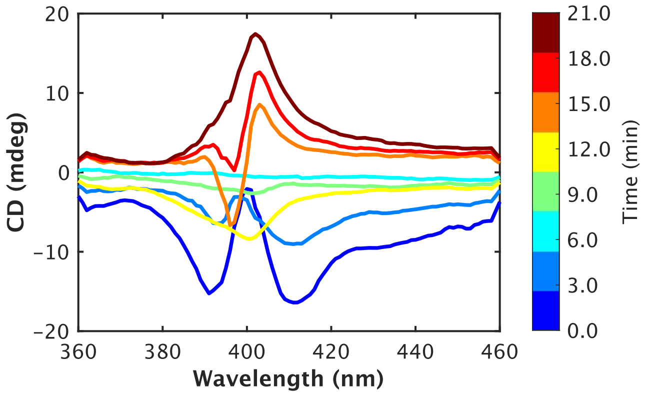

Figure S4. Time-resolved CD spectra of $CsPbBr_3$ NCLs synthesized with racemic MBA ligand, acquired with a fast scan protocol (~3 min per scan) using a reduced spectral window (360-460 nm).

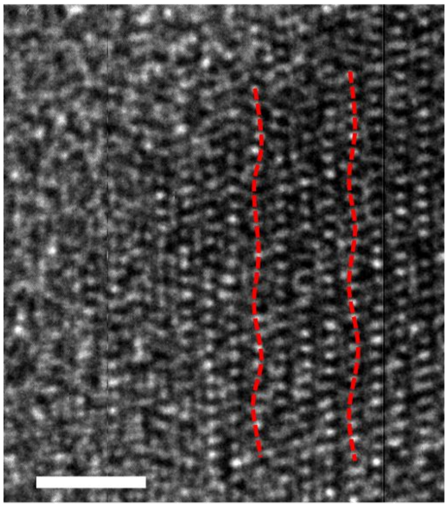

Figure S5. HRTEM of the $CsPbBr_3$ QDs (with MBA) with distorted lattice (CD peaks at 430 nm). The scale bar is 2 nm.

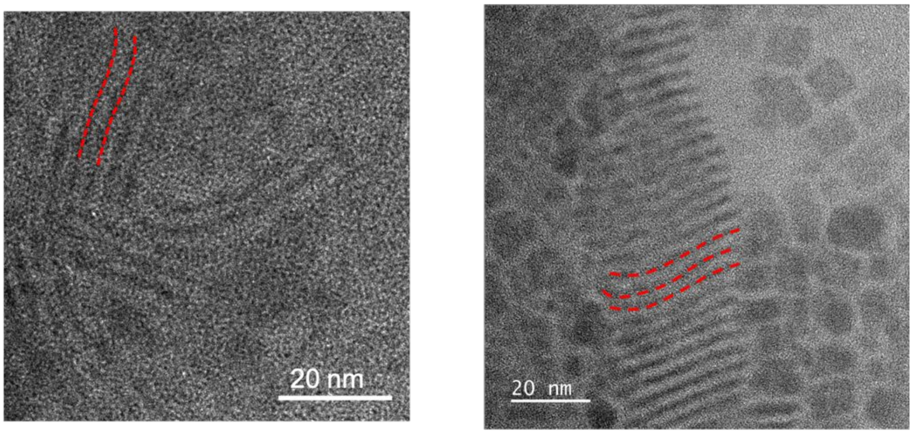

Figure S6. HRTEM of the bent, self-assembled $CsPbBr_3$ NWs (CD peaks at 430 nm).

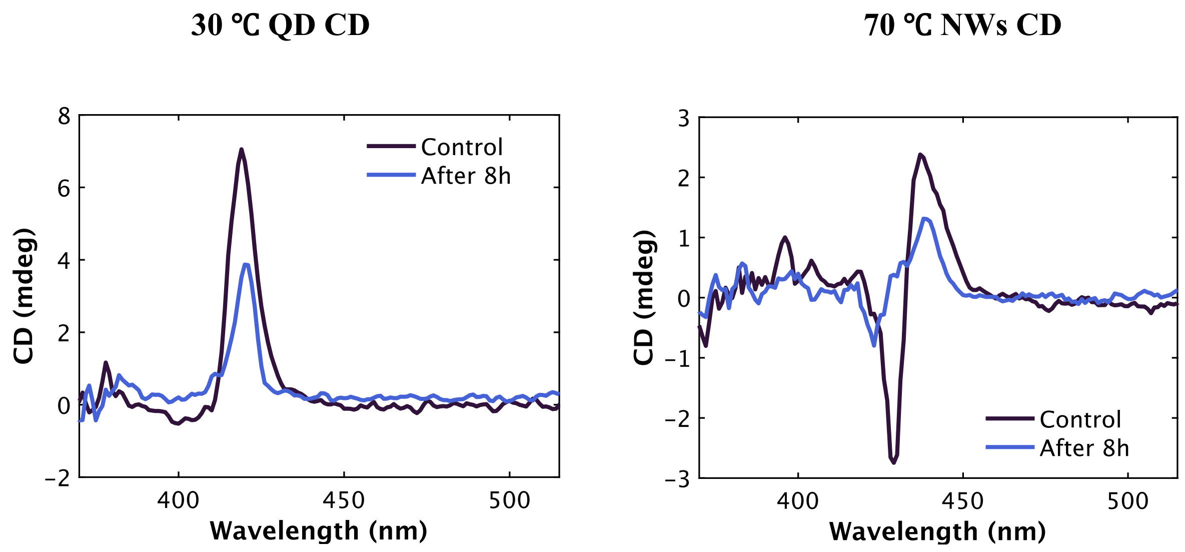

Figure S7. Stability of the CD signals of the synthesized QDs (30 °C) and NWs (70 °C). Control samples refer to the 0 h sample.

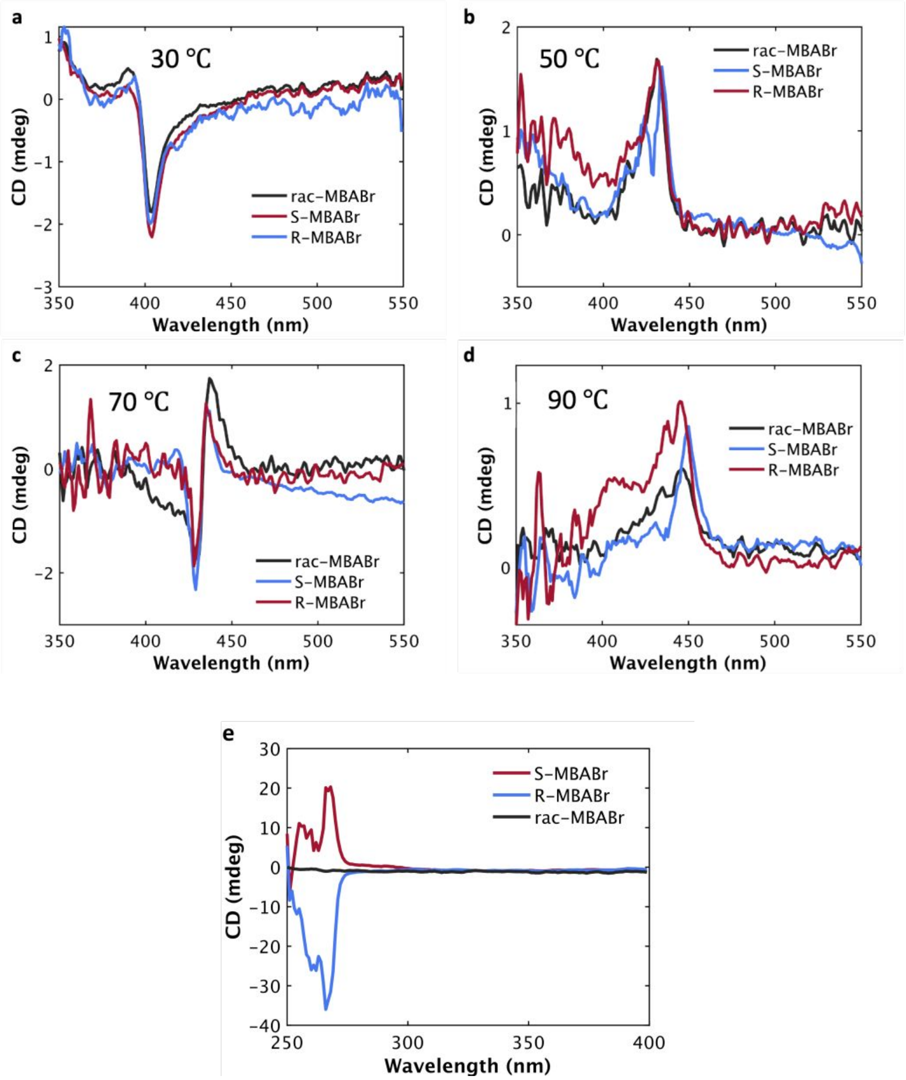

Figure S8. CD signals of the $CsPbBr_3$ nanostructures synthesized at (a) 30 ℃, (b) 50 ℃, (c) 70 ℃, and (d) 90 ℃ with rac-MBABr, S-MBABr and R-MBABr. (e) Enantiopure and racemic MBABr salt CD signal.

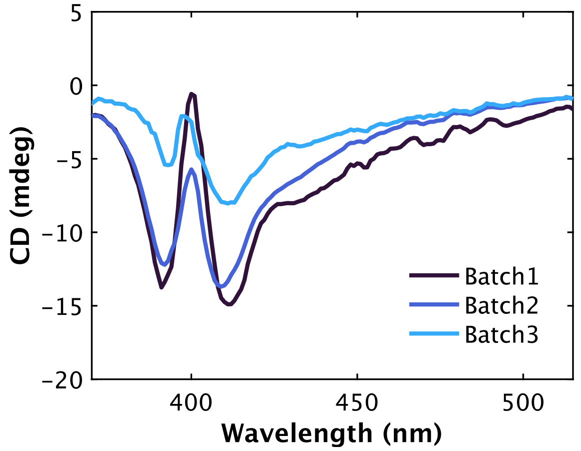

Figure S9. Batch difference for synthesized NCL CD signals

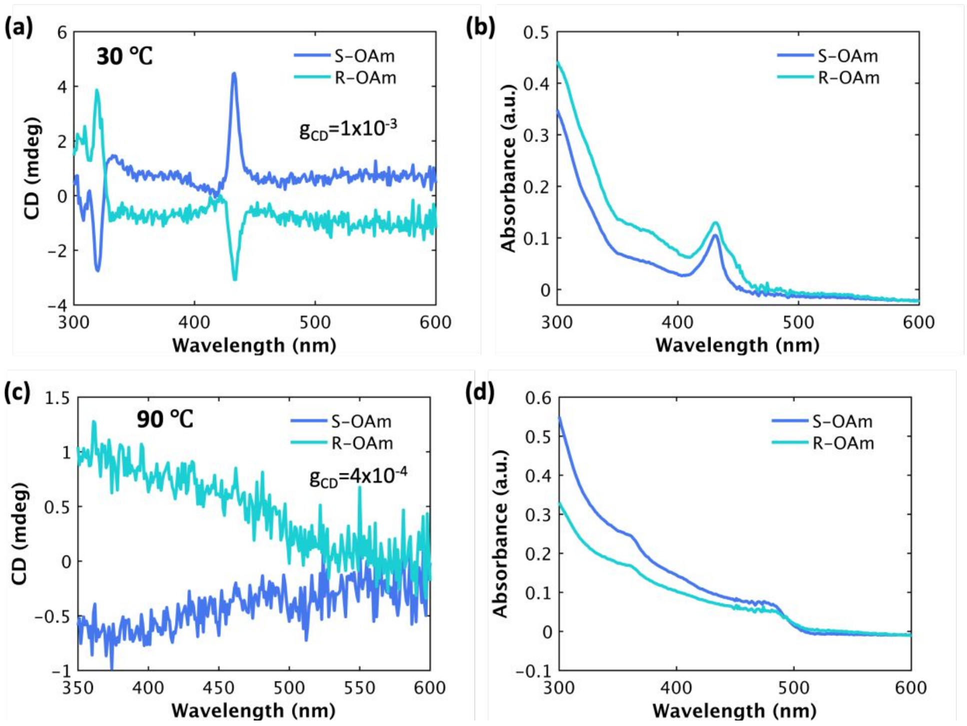

Figure S10. CD and absorption spectra of CsPbBr3 NCs synthesized using a non-aromatic chiral steric ligand, (R)- and (S)-methyloctylamine at 30 °C and 90 °C.

MBABr Concentration-Dependent Analysis

We examined the impact of MBABr amount on the emission and chiroptical properties of $CsPbBr_3$ nanostructures using PL spectra (Figure S11), CD spectra (Figure S12), and the dissymmetry factor (g_{CD}). At 30 mg MBABr (Figure S11a), steric effects were too weak to stabilize nanoclusters, leading to immediate QD formation with a 435 nm emission peak at 30 ℃. This suggests that insufficient MBA prevents the formation of nanoclusters. At 60 mg MBABr (Figure S11b), both the smallest NCLs (30 ℃) and largest NCs (90 ℃) were observed, indicating a balanced steric influence. In contrast, at 90 mg MBABr (Figure S11c) and 90 ℃, the spectrum under this condition shows PL (~470 nm) primarily from QD with only a faint shoulder at ~500 nm, indicating that NC formation is significantly suppressed but not entirely absent. These results confirm that MBA preferentially binds to smaller NCLs, slowing QD ripening.

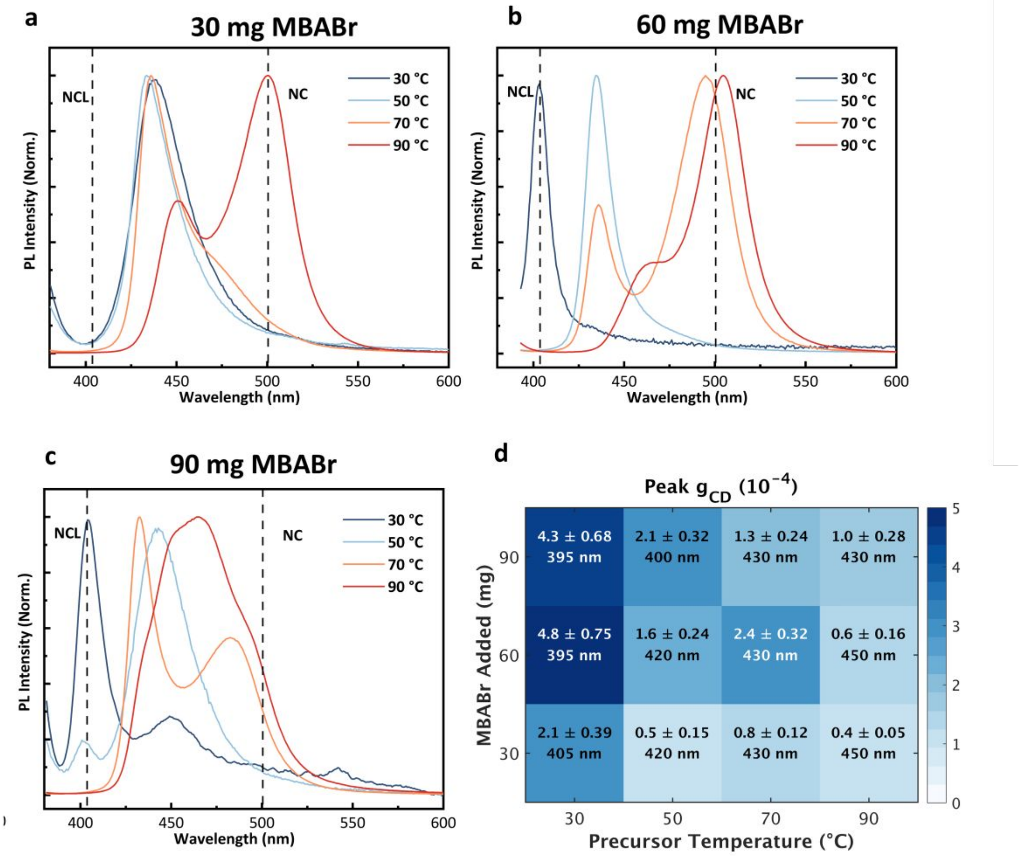

Figure S11. (a-c) PL spectra under different precursor temperatures and different amounts of MBABr. Spectra were taken 5 min after the injection. (d) MBABr concentration-dependent peak g_{CD} values at different reaction temperatures. The matrix highlights the peak g_{CD} values and their corresponding wavelengths after 30 min evolution. Error bars represent the standard deviation calculated by three different batches.

We also assessed MBA-dependent chiroptical intensity by calculating the g_{CD} values for those concentrations. We found that 60 mg MBABr tends to give the strongest CD signals at 30 °C, while 90 mg yields a comparable but not greater signal (Figure S11d), suggesting an optimal concentration around 60 mg for this system. Increasing MBABr beyond this may slightly reduce the chiral signal intensity

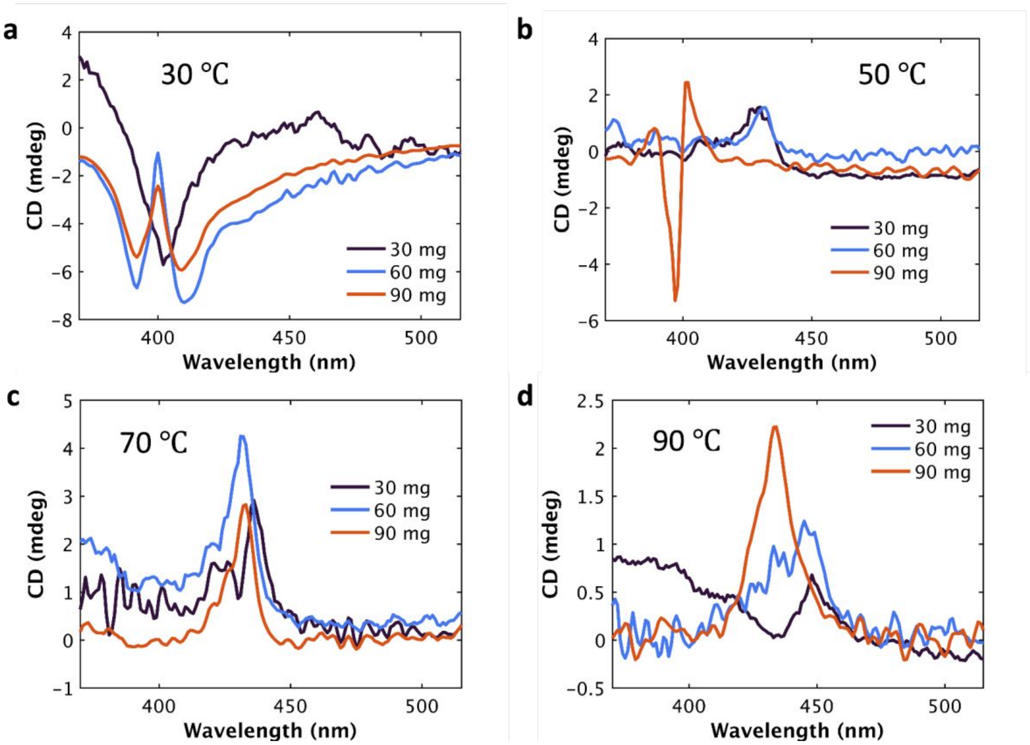

Figure S12. CD signals of the $CsPbBr_3$ nanostructures synthesized at (a) 30 ℃, (b) 50 ℃, (c) 70 ℃, and (d) 90 ℃ with different amounts of rac-MBABr. CD signals were recorded 5 minutes after the precursor injection.

Reference

(1) Jiang, Y.; Sun, C.; Xu, J.; Li, S.; Cui, M.; Fu, X.; Liu, Y.; Liu, Y.; Wan, H.; Wei, K.; et al. Synthesis-on-substrate of quantum dot solids. *Nature* **2022**, *612* (7941), 679-684.

(2) Hafner, J. Ab-initio simulations of materials using VASP: Density-functional theory and beyond. *Journal of Computational Chemistry* **2008**, *29* (13), 2044-2078.

(3) Hammer, B.; Hansen, L. B.; Nørskov, J. K. Improved adsorption energetics within density-functional theory using revised Perdew-Burke-Ernzerhof functionals. *Physical Review B* **1999**, *59* (11), 7413-7421.

(4) Grimme, S.; Ehrlich, S.; Goerigk, L. Effect of the damping function in dispersion corrected density functional theory. *Journal of Computational Chemistry* **2011**, *32* (7), 1456-1465.

(5) Tamtaji, M.; Goddard, W. A.; Chen, G. High-throughput screening of mechanically interlocked Catenane metal complexes for enhanced electrocatalytic activity. *J. Mater. Chem. A* **2024**, *12* (48), 33948-33957.

(6) Choi, J. K.; Haynie, B. E.; Tohgha, U.; Pap, L.; Elliott, K. W.; Leonard, B. M.; Dzyuba, S. V.; Varga, K.; Kubelka, J.; Balaz, M. Chirality Inversion of CdSe and CdS Quantum Dots without Changing the Stereochemistry of the Capping Ligand. *ACS Nano* **2016**, *10* (3), 3809-3815.

(7) Marenich, A. V.; Cramer, C. J.; Truhlar, D. G. Universal Solvation Model Based on Solute Electron Density and on a Continuum Model of the Solvent Defined by the Bulk Dielectric Constant and Atomic Surface Tensions. *The Journal of Physical Chemistry B* **2009**, *113* (18), 6378-6396.

(8) Tamtaji, M.; Cai, S.; Wu, W.; Liu, T.; Li, Z.; Chang, H.-Y.; Galligan, P. R.; Iida, S.-i.; Li, X.; Rehman, F.; et al. Single and dual metal atom catalysts for enhanced singlet oxygen generation and oxygen reduction reaction. *Journal of Materials Chemistry A* **2023**, *11* (14), 7513-7525.

(9) Zhou, L.; Martirez, J. M. P.; Finzel, J.; Zhang, C.; Swearer, D. F.; Tian, S.; Robatjazi, H.; Lou, M.; Dong, L.; Henderson, L.; et al. Light-driven methane dry reforming with single atomic site antenna-reactor plasmonic photocatalysts. *Nature Energy* **2020**, *5* (1), 61-70.

(10) Tamtaji, M.; Peng, Q.; Liu, T.; Zhao, X.; Xu, Z.; Galligan, P. R.; Hossain, M. D.; Liu, Z.; Wong, H.; Liu, H.; et al. Non-bonding interaction of dual atom catalysts for enhanced oxygen reduction reaction. *Nano Energy* **2023**, *108*, 108218.

(11) Badran, H. M.; Eid, K. M.; Ammar, H. Y. DFT and TD-DFT studies of halogens adsorption on cobalt-doped porphyrin: Effect of the external electric field. *Results in Physics* **2021**, *23*, 103964.
